# Nutritional constituents of mulberry and their potential applications in food and pharmaceuticals: A review

**DOI:** 10.1016/j.sjbs.2021.03.056

**Published:** 2021-03-31

**Authors:** Bisma Jan, Rabea Parveen, Sultan Zahiruddin, Mohammad Umar Khan, Sradhanjali Mohapatra, Sayeed Ahmad

**Affiliations:** a Department of Food Technology School of Interdisciplinary Science & Technology, Jamia Hamdard, New Delhi, India; b Department of Biosciences, Human Genetics and Laboratory, Jamia Milia Islamia, New Delhi, India; c Bioactive Natural Product Laboratory, Department of Pharmacognosy and Phytochemistry, School of Pharmaceutical Education & Research, Jamia Hamdard, New Delhi, India; d Department of Pharmaceutics, School of Pharmaceutical Education & Research, Jamia Hamdard, New Delhi, India

**Keywords:** Mulberry, Cosmetics, Nutraceuticals, Functional food, Value addition

## Abstract

Mulberry is a fast growing deciduous plant found in wide variety of climatic, topographical and soil conditions, and is widely distributed from temperate to subtropical regions. Due to presence of valuable phytochemical constituents, mulberry as a whole plant has been utilized as a functional food since long time. Mulberry fruits are difficult to preserve as they have relatively high water content. Therefore for proper utilization, different value-added products like syrups, squashes, teas, pestil sand köme, pekmez (turkuish by-products), yogurts, jams, jellies, wines, vinegar, breads, biscuits, parathas, and many more are made. In overseas, these value-added products are commercially sold and easily available, though in India, this versatile medicinal plant is still missing its identity at commercial and industrial scale. Leaves of mulberry are economically viable due to their important role in the sericulture industry since ancient times. Mulberries or its extracts exhibit excellent anti-microbial, anti-hyperglycaemic, anti-hyperlipidemic, anti-inflammatory, anti-cancer effects and is used to combat different acute and chronic diseases. Different parts of Morus species like fruits, leaves, twigs, and bark exhibit strong anti-tyrosinase inhibition activity that makes it a suitable candidate in cosmetic industries as a whitening agent. The current review provides a comprehensive discussion concerning the phytochemical constituents, functionality and nutraceutical potential of mulberry and as a common ingredient in various cosmetic products.

## 1 Introduction

**Fig. 1:**
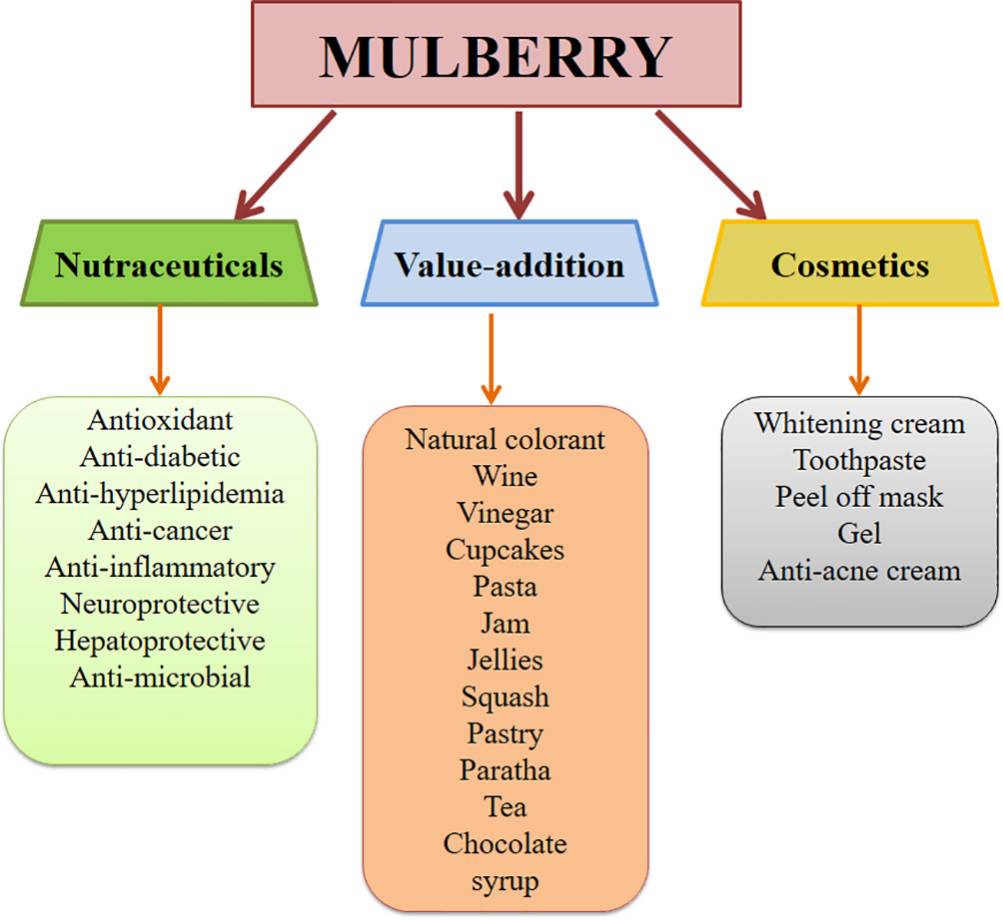
Diverse application of mulberries in multiple areas.

Mulberry belongs to the Morus genus of Moraceae family and is dispersed extensively in diverse climatic and environmental circumstances ranging from tropical to temperate. Moraceae, also known as the mulberry or fig family, is a family of flowering plants of more than twenty-four species with one subspecies and at the minimum hundred identified varieties. The term Morus is derived from the Latin word ‘mora’, which means delay, most likely because of the slow development of its buds. It is an economical and widespread woody plant and has an enormous economic value other than sericulture leading to its several unique and special features. *Morus alba* (white mulberry), *Morus nigra* (black mulberry) and *Morus rubra* (red mulberry) are all commonly accepted worldwide species of genus Morus as they exhibit maximum medicinal properties. Amongst all the species, *M. alba* is a dominant species (Ercisli and Orhan, 2007). Roots, leaves, bark, stem twigs, and fruits of mulberry possess valuable bioactive constituents that can be explored in food, health care, and cosmetic industries. Conventionally, it is believed that fruits of mulberry, particularly black and red varieties are advantageous to the human body (Ercisli and Orhan, 2007). Almost all varieties of mulberry plant are traditionally recognized in Unani, Ayurveda, and Chinese systems of medicine with several pharmacological properties. Fruits of *M. nigra* are among the important constituents of Unani medicine known as *Tutiaswad*, which is believed to have anti-cancerous activities (Nursalam, 2016). In India, mulberry is known as “KalpaVruksha” since all parts of the plant are used for various purposes and its fruit is commonly named as toot and shahtoot (King's or “superior” mulberry). Chinese utilizes mulberry fruit as a natural medicine to strengthen the joints, lower the blood pressure, treat fever, protect liver damage, and assist discharge of urine. Its fruits, leaves, and barks in traditional Turkish folk medicine have been utilized as an anti-fever, an expectorant, assists in the discharge of urine, to lower blood pressure, as a folk remedy to treat dental diseases, in dysentery, as a de-worming agent, laxative, anthelmintic, odontalgic, treat diabetes, hypertension, arthritis, and anaemia (Özgen et al., 2009). Azerbaijan people utilize *M. nigra* fruits in the treatment of ailments like gall bladder, liver, and heart diseases (Farid Alakbarli and Iskandar Aliyev, 2000). The presence of valuable constituents in mulberry leaves and fruits makes the plant suitable to be placed in the category of functional foods that are useful to human health in addition to its basic nutritional function (Kadam, 2019). Leaves of *M. nigra* are commonly used by women during menopause as a replacement for the conventional hormonal substitute therapy, with a similar effect to that obtained by estrogenic use (de Queiroz et al., 2012). Furthermore, its fruits, roots, and leaves extracts can be utilized in cosmetics globally and is a commonly used constituent in many dermatological creams, bath gels, and many more owing to its exceptionally high radical scavenging potential. As stated by the Ministry of Health of China in 1985, *M. alba* was recorded as the first medicinal and edible fruit (Yuan and Zhao, 2017), and its leaves and fruits were considered not only food but also as drugs (Wang et al., 2014). Mulberry fruits are soft and delicate, and harvesting season lasts for a month usually from May-June in most parts of the world and the best growing temperature is between 24 and 28 °C (Sharma and Zote, 2010, Dhiman et al., 2020). To properly utilize the mulberry or enhance the storage life, maintain nutritional and organoleptic qualities, and to minimize the waste, the possibility of introducing mulberry as a functional food and nutraceutical is a need of the hour that many researchers are looking forward to. Nonetheless, being exceptionally good in nutrients and comparatively low in fats can be considered a good choice for healthy consumers. An overview of multifunctional role of mulberry is shown in Fig. 1**.**

Based on the existing literature on the mulberry, it will be interesting to review the effectiveness of this multifunctional plant in attributing different functional properties, when being incorporated in several food products and to explore its nutraceutical applications and dermatological role with special emphasis on tyrosinase inhibition activity.

The current review attempted to provide a holistic insight into the nutraceutical potential of Morus in preventing various health-related issues and to investigate the possibilities of using mulberry as a functional food with some formulations and as a common ingredient in many cosmetics.

## 2 Botanical description of Morus

**Fig. 2:**
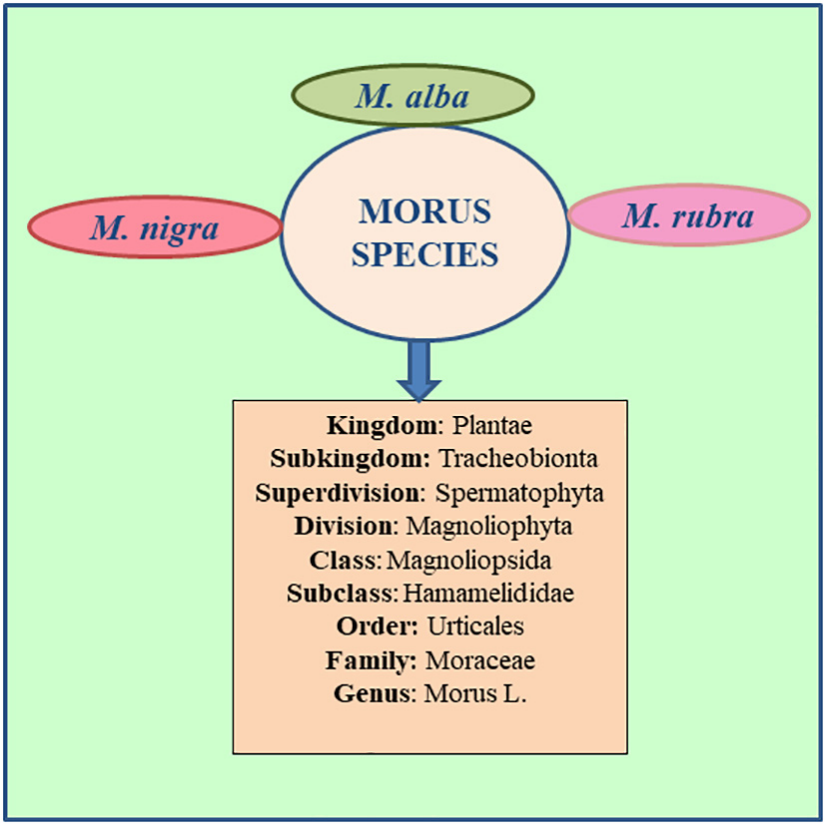
Scientific classification of mulberry.

Mulberry is typically a deciduous or medium sized woody perennial tree having upright fissured bark and cylindrical stem with a milky sap growing upto 10–13 m tall (Singh et al., 2010, Rahman and Khanom, 2013). Leaves of mulberry vary in shape and size, usually range from 5 to 7.5 cm long and 6–10 cm wide and are mostly deeply lobed, margins serrate, shortly acuminate, apex acute or, base cordate or truncate; 3 basal nerves, lateral nerves forked near the margins. Flowers are yellowish green in colour with chromosome number 2n = 28. Female spikes are ovoid and stalked while as male spikes (catkins) are cylindrical and broad. Male catkins tend to be longer than female catkins. Botanically, mulberry fruit is precisely a cluster of small fruits that are organised longitudinally around the central axis similar to that in blackberry or loganberries. Its fruit or syncarp comprises of numerous drupes that are enclosed in a fleshy perianth, ovoid or sub globose, upto 5 cm long, white to pinkish white, purple or black when ripe (Anonymous, 2001). In terms of morphology and growth habits, mulberry ovary is unicellular with a bifid stigma and analogous to that of other fleshy drupaceous fruits. Scientific classification of Morus species is given in Fig. 2.

## 3 Dietary importance of Morus

**Fig. 3:**
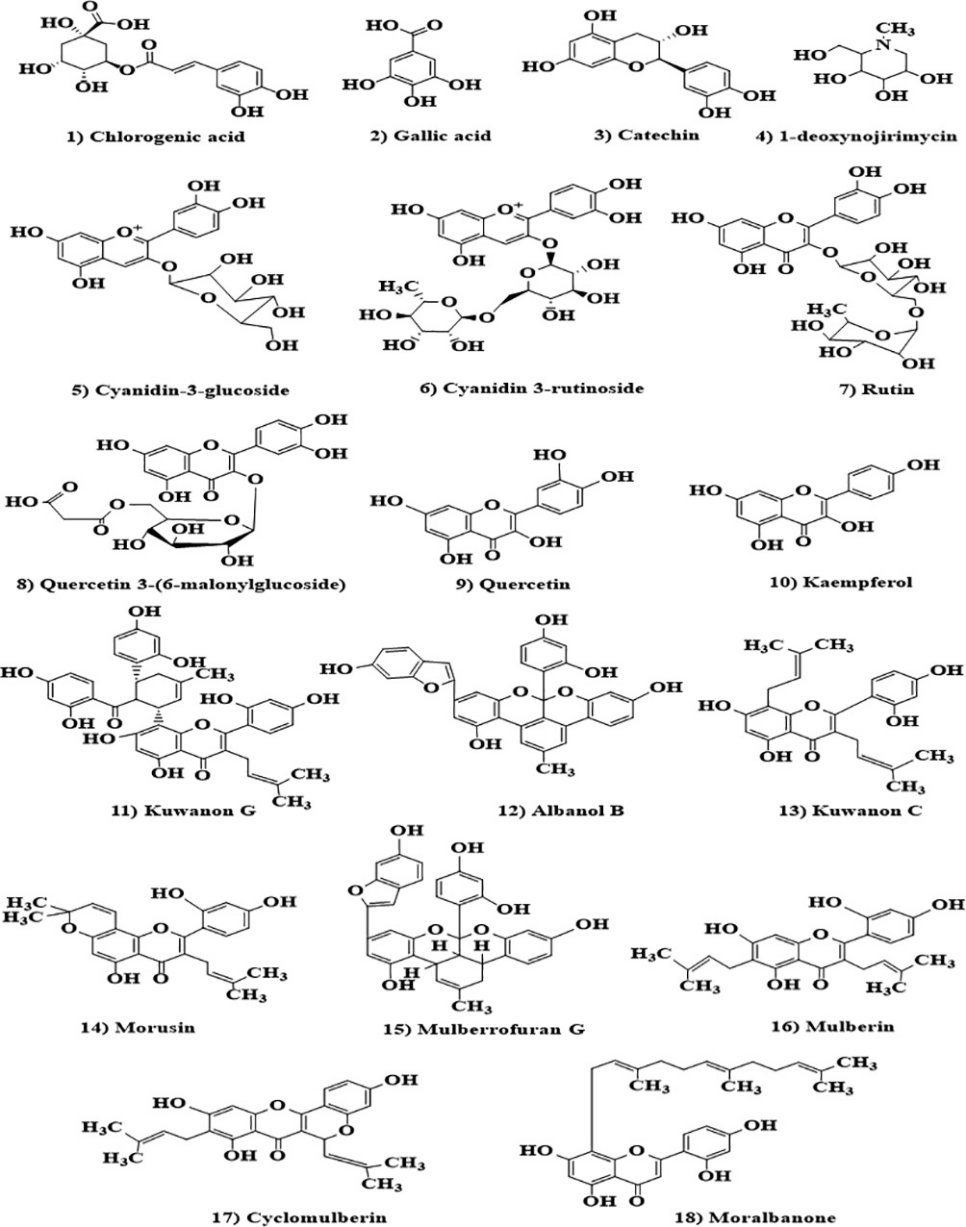
Chemical structures of some important metabolites of mulberries.

Fully ripened mulberry fruit has a wonderful mouth-watering taste with a good aroma and flavour. It is appreciated for direct consumption and for making value-added products. Mulberry fruits are recognized for the well-being of human beings due to their high nutritional significance (Sengül et al., 2005). Additionally, mulberry fruits hold a diversity of nutrient elements that play a vital function in human metabolism (Akbulut and Özcan, 2009). *M. alba* fruit is a good resource of carbohydrate, lipid, protein, vitamins, minerals, and fibers. The quantity of protein in fresh *M. alba* fruit is greater than that of raspberries (Rao and Snyder, 2010)and strawberries (Giampieri et al., 2012)and comparable to blackberries, (Kaume et al., 2012)whereas the anthocyanin content is higher than blackberry, blueberry, blackcurrant, and redcurrant (Veberic et al., 2015). *M. alba* fruit contains both essential and non-essential amino acids. Essential amino acid /total amino acid ratio is 42 percent, which is almost equal to certain protein-rich foods such as fish and milk (Jiang and Nie, 2015). Hence, can be considered as an excellent protein source. Chemical structures of some important metabolites are given in Fig. 3. Each variety of Morus species contains a significant amount of vitamin C, however among all varieties *M. nigra* contains the maximum quantity. The ascorbic acid content in *M. alba* and *M. nigra* is 15.81 and 12.81 mg/100g, respectively of fresh fruit weight (Eyduran et al., 2015). Mulberries also contain some important alkaloids that activate macrophages by stimulating the immune system and hence safe guard the human body against health threats (Kim et al., 2013). The most important alkaloids isolated from mulberry leaves are 1-deoxynojirimycin (DNJ),1,4-dideoxy-1,4-imino-D-ribitol, and 1,4-dideoxy-1,4-imino-D-arabinitol (Li et al., 2013, Sharma et al., 2010, Li et al., 2011). Primary sugars present in mulberry are fructose and glucose, which increase with ripening (Gundogdu et al., 2011, Mahmood et al., 2012, Eyduran et al., 2015). Amongst the widely recognised varieties, *M. alba* has the maximum fat content of 1.10% followed by *M. nigra* with0.95% and *M. rubra*with0.85%. Oleic acid, palmitic acid and linolenic acid are the major fatty acids in mulberry fruit (Ercisli and Orhan, 2007). The sequence of fatty acids in *M. alba* fruit is polyunsaturated fatty acids (PUFA) followed by monounsaturated fatty acid (MUFA) and saturated fatty acids. Among all fatty acids, PUFA is the main fatty acid in mulberry fruits comprising at least 76.68%, which is even higher than that of strawberries (Giampieri et al., 2012, San and Yildirim, 2010). There are many organic acids present in mulberry fruits *viz* citric acid, tartaric acid, malic acid, succinic acid and fumaric acid however, malic acid is primarily found organic acid in all the species (Eyduran et al., 2015). Mulberry is also an excellent source of some important minerals particularly calcium, phosphorus, potassium, magnesium, and sodium. Nonetheless, the mineral content differs among phenotypes (Gungor and Sengul, 2008). Although *M. alba, M. nigra* and *M. rubra* belong to same genus however, there are still some differences in their physiochemical parameters. A comparative study of varied physiochemical parameters of mulberry varieties (*M. alba, M. nigra* and *M. rubra*) is given in Table 1.

**Table 1:** Physicochemical paramter of different varieties of mulberry.

**Physiochemical Properties**	***Morus alba***	***Morus nigra***	***Morus rubra***	**Reference**
Moisture (%)	71.5	72.6	74.6	Imran et al., 2010, Ercisli and Orhan, 2007
Protein (g^−1^100 g DW)	1.55	0.96	1.2	Koca et al., 2008
Fat (%)	1.10	0.95	0.85	Ercisli and Orhan, 2007
Fiber g^−1^100 g	1.47	11.75	–	imran et al., 2010
Ash (g^−1^100 g)	0.57	0.50	2.45	imran et al., 2010
Total dry weight	29.5	27.	24.4	Ercisli and Orhan, 2007
Ascorbic acid mg^−1^100 g	22.4	21.8	19.4	Ercisli and Orhan, 2007
Total acidity (%)	0.25	1.40	1.37	Ercisli and Orhan, 2007
pH	5.60	3.52	4.04	Ercisli and Orhan, 2007, Koca et al., 2008
Calcium (mg^−1^100 g)	152	132	132	Ercisli and Orhan, 2007
Mg (mg^−1^100 g)	106	106	115	Ercisli and Orhan, 2007
K (mg^−1^100 g)	1668	922	834	Ercisli and Orhan, 2007
Fe (mg^−1^100 g)	4.2	4.2	4.5	Ercisli and Orhan, 2007
Nitrogen (%)	0.75	0.92	0.82	Ercisli and orhan 2007
FRAP (µmol TE^−1^g fw)	4.494	12.9	6.4	Gundogdu et al., 2011
Total phenolics (mg QE^−1^100 g fresh mass)	181	1422	1035	Ercisli and Orhan, 2007
Total flavnoids (mg QE^−1^100 g fresh mass	29	276	219	Ercisli and Orhan, 2007
Total anthocyanins content C3G µg^−1^g frozen weight	911.8	719	109	Natić et al., 2015
Total antioxidant capacity (mol TE g^−1^ fw)	4.494	13.999	5.497	Gundogdu et al., 2011
Malic acid (g 100 ^g−1^ fw)	3.095	1.323	4.467	Gundogdu et al., 2011
Succinic acid (g 100 ^g−1^ fw)	0.168	0.342	0.132	Gundogdu et al., 2011
Citric acid (g 100 g^−1^ fw)	0.393	1.084	0.762	Gundogdu et al., 2011
Total organic acid (g 100 g^−1^ fw)	3.983	2.951	5.812	Gundogdu et al., 2011
Total soluble solids (%)	7.27	11.60	19.20	Aljane and Sdiri, 2016
Fructose (g 100 g^−1^ fw)	6.269	5.634	5.407	Gundogdu et al., 2011
Glucose (g 100 g^−1^ fw)	6.864	7.748	6.068	Gundogdu et al., 2011
Hunter L*	78.4	14.3	27.3	Ercisli and Orhan, 2007
Hunter a*	−13.6	7.02	8.55	Ercisli and Orhan, 2007
Hunter b*	16.2	1.72	2.02	Ercisli and Orhan, 2007

## 4 Role of Morus in food industries

The understanding of the relationship between diet and health by customers has now acquired a profound shift in eating pattern and lifestyle transformation. The advent of this consumer understanding has been one of the driving forces in production of food products that can satisfy both basic dietary requirements and health benefits. Mulberry fruits are famous throughout the world for their mouth-watering taste that makes it suitable to consume either in fresh or as an ingredient in value-added products and for culinary uses. It has gained popularity due to consumer awareness and enthusiasm for healthy and low calories foods. This has led to increased demand in food processing industries. The role of mulberry in diverse food areas is mentioned in Table 2**.** Ripened mulberry fruits are harvested by slightly shaking trees (Singhal et al., 2010). Mulberry fruit is highly perishable making it underutilized, however, there is the scope of value addition by various means. It contains health-promoting polyphenols and is consumed directly or in processed product forms such as juices, syrups, liquors, molasses, jams, wines, and soft drinks. Mulberry fruits are among the berries that can be called as superfood and can be industrially explored for diverse commercially priceless valuable edible products. Several patents have been filed on Morus species for multiple therapeutic applications such as hypoglycaemic, neurodegenerative, hypolipidemic, compounds with anti-tyrosinase inhibition and some formulated value-added products as enlisted in Table 3.

**Table 2:** Role of mulberry in different food industries and major findings.

**Application**	**Major findings**	**Reference**
Natural colorant in yogurt	Colouring potential of *M. rubra* was studied in yogurt and the colour developed by adding mulberry anthocyanins was similar to commercial brand strawberry yogurt	Byamukama et al., 2014
Antioxidant component in museli	*M. alba* fruit was incorporated in museli that resulted in significant increase in its antioxidant, and nutritional value	Kobus-Cisowska et al., 2013
Ready to serve juice	Cloudy dark purple mulberry juice containing 0.5% xanthan gum as the stabiliser had the highest levels of acceptance after storage without precipitation	Akkarachaneeyakorn and Tinrat, 2015
Wine	*M. alba* fruit was used as raw material to brew fruit wine. Phenolics present in the wines were detected by HPLC	Wang et al., 2015, Yadav et al., 2017, Kim et al., 2008
Sake	Mulberry leaves were utilized for the production of sake or rice wine by fermentation with Mauri yeast and product was rich in nutrients, amino acids, and polyphenolics	Tan and Li, 2013
Vinegar	Vinegar produced from *M. alba* exhibited powerful antioxidant potential and showed anti-microbial effects	Karaagac et al., 2016
Jelly	Anthocyanin-rich jelly was developed by adding *M. alba* fruit containing anthocyanins. The consumption of formulated functional product resulted in a significant decrease in fasting blood cholesterol and LDL in dyslipidemia patients	On-Nom et al., 2020
Syrup	*M. alba* fruit was utilized for the development of syrup and when packed in PET bottles can be stored for six months under ambient and refrigerated conditions	Thakur and Abhimanyu Thakur, 2017
Squash	*M. alb*a fruit can be utilized to develop appetizer or spiced squash after optimization and when stored in PET bottles can retain better quality attributes	Hamid and Thakur, 2017
Mixed fruit jam	Mixed fruits jam based on rosella and mulberry in the ratios of 70/30 was selected based on overall acceptability	Wongchalat and Chatthongpisut, 2016
Alcoholic beverage	*M. alba* fruit can be utilized to produce alcoholic beverages. However, it is not possible to make wine from fresh black mulberry juice due to the low alcohol level that the beverage showed after fermentation	Darias-Martín et al., 2003
Chocolate	Chocolate can be fortified with encapsulated anthocyanins from spray-dried *M. nigra* fruit waste hence, can be utilized in a better way in food and pharmaceutical industry	Gültekin-Özgüven et al., 2016
Probiotics	*M. alba* silage is a potential source for the isolation of lactic acid bacteria. In a study 38, lactic acid bacteria were isolated from mulberry silage however only four strains were capable to survive in the gastrointestinal tract	Shokryazdan et al., 2015
Pastry	*M. alba* extract along with buckwheat flour, buckwheat hulls, chokeberry, and inulin can be utilized to produce pastry with lower energy level and higher fibre content.	Komolka et al., 2016
Pasta	Enriched pasta by different formulations of *M. nigra* extract exhibited hypoglycaemic effect by decreasing the glycemic index and inhibiting α-amylase and α-glucosidase activity.	Yazdankhah et al., 2019
Minced meat	Methanolic extract of mulberry leaves increased the shelf life of minced meat	Yazdankhah et al., 2019
Cupcake	Cupcakes were prepared from concentrated paste of *M. alba* fruits with accepable and sugar beet root production while maintaining satisfactory organoleptic and physico-chemical parameters	Jabborova et al., 2020

**Table 3:** List of patents published from on mulberry with emphasis on therapeutic, cosmetic and functional applications 2011–2020.

**Patent no.**	**Publication date**	**Title**	**Purpose**
US 2011/0064866 A1	17-Mar-11	Black mulberry flavoured composition and method of preparation	*M. nigra* flavoured composition was prepared from water, tagette essence, blueberry essence, grape essence apple juice concentrate, blackberry juice concentrate, blue berry juice concentrate, and raspberry added at different times and mixing the ingredients at various times during the process
US 2013/0108567 A1	2-May-13	Skin-whitening composition for priority data external application on skin containing extracts from paper mulberry flowers and fruits	An extract of the flower and fruit of paper mulberry inhibits melanin production, therefore, has an excellent skin whitening effect
US 2014/0356468 A1	4-Dec- 2014	Composition containing paper mulberry extracts	Paper mulberry extract possessing cosmetic composition has an important role in many skin related functions like enhancement of skin moisturization, inhibition of skin aging, alleviation of inflammation, antibacterial activity, pore size reduction, sebum control, skin complexion improvement, decomposition of subcutaneous fat, stimulation of melanin synthesis, and gray hair prevention
US 9,040,106 B2	26-May-15	Pharmaceutical composition for preventing or treating diabetic erectile dysfunction comprising C3g or extract of mulberry containing C3g	Cyanidin-3-O-3-d-glucopyranoside present in mulberry has an ameliorating effect particularly for preventing or treating diabetic erectile dysfunction
US 9,066,960 B2	30-Jun-15	Use of the effective fraction of alkaloids from mulberry twig in preparing hypoglycaemic agents	Alkaloids present in mulberry twig are 50% or more by weight in the effective fraction and are said to have hypoglycaemic agents
US 2018/0139966	24-May-18	Method for treatment of mulberry leaves and for anti -bacterial silk production	Development of intrinsically antibacterial silk directly from the silkworm, by feeding the worm on mulberry leaves or silver treated feedstuff
US 2019 / 0,314,439 A1	17-Oct- 2019	Method for suppressing obesity or development of obesity	Fermented Indian mulberry comprised of appetite-suppressing composition resulting in prevention and amelioration of obesity and other health problems as a result of reduced food intake
US 10, 588, 927, B2	17-Mar-20	Composition containing mixed extract of mulberry and *Poria cocus* peel	Treating or improving neurodegenerative disorders
US 2020/0178585 A1	11-Jun-20	Savoury concentrate with mulberry fruit extract	Significantly be used in the preparation of starch rich food like pasta
US 2020/0197429 A1	25-Jun-20	Dietary supplement for glycemic control and diabetes prevention	Formulation containing root extract of mulberry along with some other functional extracts of astragalus root, phlorizin has glucose-lowering effect

Mulberry may be well exploited in fruit and vegetable industries for making, marmalade, fondant jams, jellies, cakes, breads, parathas, fruit teas, fruit drink pulp, fruit wine, fruit sauce, fruit powder, and chocolate, due to high sugar content. Moreover, these fruits are either used in dried, frozen, or fresh forms in the food industry to yield different syrups, amaretto or vermouth wine, tonic wine, and vinegar. Mulberry seeds can also yield oil. ‘Pestil’ and ‘köme’ are the famous traditional Turkish foods, which are prepared from a mulberry, walnut, hazelnut, honey, and flour mixture (Oktay, 2013, Sengül et al., 2005, Ercisli and Orhan, 2007). Persian utilize mulberries for making jellies, desserts and sauces. Unripe and immature fruits are utilised for chutney preparations (Jalikopa et al., 2011). Pure and fresh mulberry fruit juice under cold storage environment remains fresh for a duration of three months and bottled juice can stay fresh at ambient temperature for a period of six months to a year. This mulberry juice aids in keeping healthy and smooth skin, prevents irritations, inflammations and throat infections, and has also has laxative properties (Buhroo et al., 2018). It is also used as medicine to reduce fever, cold, diarrhoea, endemic, malaria, and amoebiosis (Kumar and Chauhan, 2008). In China, mulberry is usually available in the form of a paste famous as sangshengao. This paste is dissolved in warm water to make tea that improves kidney and liver functions and enhances the vision and hearing. Chinese people also take young leaves and tender shoots of mulberry as vegetables in some specified regions. Iranian people utilize dehydrated mulberries as a sweetening agent in black tea.

Mulberry fruits can be used to make syrup due to their high sugar content. Mulberry syrup is widely consumed as processed mulberry products in Vietnam. In syrup production, usually fresh mulberry fruit is mixed with cane sugar in a ceramic or glass bottle and can be stored for at least 2 weeks (Quang Trung et al., 2018). Mulberry fruit powder prevents aging of the skin by disturbing the formation of free radicals in cells. It also manages good cholesterol in the human body and balances the absorption of carbohydrates (Liu et al., 2009). Mulberry wine, which is sweet and sour can be produced from over-ripened mulberry fruits (Feng et al., 2015). This functional wine aids in removing unwanted faecal impurities from the body and may help in making the body lean and function as medication to tonify the masculine weakness after diseases. Mulberry wine is well-known in Europe as a name lady’s drink. In countries like Armenia, Azerbaijan, and Georgia, mulberry is a famous liquor known as Tut araghi*.* This drink is placed among the national Azerbaijani type of vodka and little quantity of it protects from stomach and cardiovascular diseases (Farid Alakbarli and Iskandar Aliyev, 2000). Mulberry fruit is a concentrated source of anthocyanins mainly cyanidin-3-glucoside (C3G) and cyanidin-3-rutinoside (C3R)that can be utilized as a natural colorant in food industries (Du et al., 2008, Aramwit et al., 2010, Zhang et al., 2011). Recently, the effect of polyphenols in mulberry juice on the oxidation stability and functional properties of myofibrillary and sarcoplasmic proteins in dried minced pork slices during storage and processing was studied. Structural stability was improved to a greater extent by reducing protein aggregation, carbonyl accumulation, and transformation of SH group into S-S group in pork slices (Cheng et al., 2020). Similarly, mulberry extracts demonstrated a protective effect on protein oxidation of dried-minced pork slices (Cheng et al., 2018). Mulberry leaves can be incorporated into wheat flour to make paratha with different mix ratios. The optimized mix has storage stability of two weeks at room temperature and does not exert any adverse effect on the growth of visceral organs of rats (Srivastava et al., 2003).

One of the special caffeine-free teas made from mulberry leaves is mulberry tea. It is popular in China, Thailand, Japan, and Korea, where it has been used in conventional medicine since ages. Itis famous for enhancing liver and kidney functions, sharpening hearing, and brightening the eyes. This tea also relieves cough, cold, and throat infections, and also inhibits cholesterol oxidation, thereby freeing the arteries from fat deposition, hence avoiding artery hardening (Yu et al., 2018). Because of its anti-diabetic and cholesterol-lowering properties, this functional tea is a very popular drink. In case of a throat infection, a decoction of leaves is often used as a gargle (Buhroo et al., 2018).

Nutritionists and health experts have recently placed *M. alba* tea in the list of superfoods especially in European countries (Natić et al., 2015, Krishna et al., 2020). Mulberry tea, particularly from *M. alba,* health benefits are largely due to its naturally occurring ingredient, DNJ. It holds anti-diabetic effects, due to its ability to decrease carbohydrate absorption and helps to regulate the level of blood sugar in diabetes. However, it is necessary to steep it for a suitable period to obtain as many of the benefits of mulberry tea as possible. Brewing of one gram of mulberry leaves in 100 ml of water for 3–5 min at 98 °C will lead to effective inhibitory activity against certain enzymes such as maltase (Hansawasdi and Kawabata, 2006).

Thus, it is evident from the above studies that mulberry can be effectively used in the food and beverage industries as an interesting raw material being exceptionally rich in antioxidants with a strong nutritional profile.

## 5 Anti-tyrosinase properties of Morus for cosmetic application

Use of tyrosinase inhibitors is becoming increasingly important in the cosmetic industry due to their skin-whitening effects. Tyrosinase is a copper-containing primary regulatory multifunctional enzyme that is responsible for melanin biosynthesis and determines the colour of the skin and hence it can be used as a whitening agent. Excessive melanin deposition induces numerous dermatological disorders, such as melasma and age spots (Mukherjee et al., 2018). Roots and twigs of Morus could be utilized as promising natural agents to counteract tyrosinase activity in cosmetics given in Table4**.**

**Table 4:** Reported tyrosinase inhibitory phytoconstituents in mulberry.

**Species/part**	**Compound**	**Extract**	**Salient findings**	**Reference**
*M. nigra*/roots	Moracin N, kuwanon H, morachalcone A, mulberrofuran G, 5′-geranyl-5′,7′,20′,4′-tetrahydroxyflavone, steppogenin-7-O-β-D-glucoside.	Ethanol	IC_50_ of Isolated compounds showed better tyrosinase inhibitory activities than kojic acid	Zheng et al., 2010
*M. alba/* twig	Morusone, steppogenin, 2, 2,2′,4′,tetrahydroxychalcone, morachalcone,oxyresveratrol and moracin	Ethanol	Potential natural tyrosinase inhibitors in cosmetics as skin-whitening agents	Zhang et al., 2016
*M. alba*/twigs/roots	Maclurin and morin	Ethanol	The anti-tyrosinae activity of twigs was better than roots	Chang et al., 2011
*M. alba*/leave	Mulberroside F	Hydroalcoholic	Isolated compound showed inhibitory effects on tyrosinase activity and on the melanin formation of melan-a cells	Lee et al., 2002
*M. alba*/leave	Moracin J	Ethanol	The isolated compound could be utilized to inhibit melanin production through the regulation of melanogenesis-related protein expression	Li et al., 2018
*M. alba*/root	Oxyresveratrol, oxyresveratrol-3-O-glucoside, and mulberroside	Ethanol	Inhibited the pigmentation in guinea pig skin when applied topically without causing any eye irritation and skin sensitization	Park et al., 2011
*M. alba*/wood	Oxyresveratrol, *trans*-dihydromorin, and 2,4,3′ -trihydroxydihydrostilbene	Methanol	Suppressed melanogenesis in the zebrafish model hence can be used in treating the disorders associated with melanin pigment	Chaita et al., 2017
*M. australis/*stem	Austraone	Ethanol	Isolated new compound exhibited moderate tyrosinase inhibitory activity	Zheng et al., 2012

Most Asian countries use *M. alba* (leaves, fruits, root bark and branches) as an ingredient in cosmetics (Li et al., 2018). In a study, ethanolic extract of *M. alba* fruit was utilized to develop an emulsion-based cream to study its clinical effect on skin melanin, erythema, and moisture content for eight weeks. The formulated cream significantly decreased melanin content without causing any type of skin irritation (Akhtar et al., 2012). In other research, betulinic acid (C_30_H_48_O_3_) was isolated from *M. alba* (hexane extract of stem and root bark)which can be utilized as a whitening agent owing to its tyrosinase inhibitory activity (Nattapong and Omboon, 2008). Ethanolic extract of *M. nigra* exhibits excellent tyrosinase inhibition activity and also be utilised for the formulation of peel-off mask and for acne treatment (Budiman et al., 2017a, Budiman et al., 2019)*.*

Mulberries can help to mitigate skin problems such as reduction in spots and blemishes appearing with age and inhibition of free radical linked oxidative activity thereby bestowing a healthier and shiny appearance to skin and hair. Hence from the above discourse, it is clear that different parts of Morus exhibit excellent tyrosinase inhibition activity, and hence can be included as a necessary component of cosmetic products and de-pigmentation agents for the treatment of hyperpigmented disorders.

## 6 Nutraceutical applications of Morus

Nutraceuticals have various therapeutic properties that are primarily due to their chemical structure, anti-oxidant, anti-diabetic, anti-hypertensive, hypo-cholesterolemic, anti-microbial, hepatoprotective properties, and many more. Some of the traditional formulations containing mulberry as an ingredient are listed in Table 5 and Table 6 summarizes some findings on nutraceutical applications of Morus plant. The current available literature on the nutraceutical ability of Morus species to improve human health and well-being is presented in this section.

**Table 5:** Available traditional formulation containing mulberry extract as one of ingredient.

**Brand name**	**Formulation name**	**Composition**	**Function**
Green silk	Green silk formula 1	*M. alba* extract, wolfberry extract, milk thistle extract, chicory root extract, safflower extract, nettle extract, cayenne fruit extract	Lowers blood sugar, improves cholesterol and weight loss
Hamdard Laboratories	Sharbat Toot Siyah	*M. nigra* with sugar	An effective herbal remedy in swelling and pain of throat (Pharyngitis)
Ahana Nutrition	White mulberry leaf extract	*M. alba* leaf extract, *Garcinia cambogia*, green coffee bean, african mango extract, cinnamon	Slows down the build of cholesterol plaque around arteries and minimizes the progression of atherosclerosis
BioGanix	White mulberry leaf extract	*M. alba*, vegetable cellulose	Maintains healthy blood sugar levels, curbs appetite
Vox nutrition	White mulberry leaf pure	*M. alba* leaf extract standardized to 1% alkaloids, 15% quercetin and isoquercetin and inactive ingredient cellulose	Weight loss and craving control
Immortalitea	White mulberry leaf	100% *M. alba* leaf	Caffeine-free weight loss tea
Nature’s	Max slim white mulberry blend	*M. alba* extract 500 mg with *Garcinia cambogia*, green coffee bean, African mango	Sugar blocker and appetite suppressant diet pill
Naturesque	White mulberry leaf extract	*M. alba* leaf extract 1000 mg, vegetable cellulose (capsule), microcrystalline cellulose	Helps to reduce sugar and carb cravings, help lower blood sugar, supports cardiovascular health
Hamdard Laboratories	Sualin	*M. nigra* 50 mg, liquorice extract 8.571 mg, *Adhatoda vasica* extract 5.714 mg, *Ocimum basilicum* extract 5.714 mg, menthol 0.00171 mg, oil anise 0.00054 µl, oil eucalyptus 0.00053 µl, oil pine 0.00043 µl, oil cubeb 0.00016 µl and oil cinnamon 0.00011 µl	Sualin tablet is used to treat sore throat, cold, cough and bronchitis

**Table 6:** Nutraceutical applications of mulberry.

**Biological activity**	**Extract**	**Species/Part**	**Salient findings**	**Reference**
Antioxidant	Ethyl acetate	*M. alba*/ fruit	*M. alba* fruit extract showed excellent *in vitro* radical-scavenging activities against DPPH and superoxide anion radicals and increase antioxidant enzymatic activities like SOD, CAT, and GSH-Px in STZ-induced mice	Wang et al., 2013
	Ethanol	*M. alba*/leave	Moracin extracted from *M. alba* leave exhibited antioxidant activity better than resveratrol	Tu et al., 2019
	Ethanol	*M. nigra*/fruit	Polysaccharides in *M. nigra* fruit exhibited the strongest protective effect on H_2_O_2_-induced oxidative injury in PC12 cells	Wang et al., 2018
	Ethanol	*M. alba/* stem	*M. alba* stem extract increase superoxide and NO scavenging activity as well as iron reducing capacity *in vitro*	Pham et al., 2017
	Powdered leaves mixed with diet.	*M. indica*/leave	*M. indica* leaf powder resulted in improvement of antioxidant enzymes viz., GPx glutathione reductase (GR), glutathione-S-transferase (GST) SOD in STZ induced wistar rats	Andallu and Varadacharyulu, 2003
	Ethanol	*M. alba*/ fruit	Flavnoids extracted from *M. alba* fruit showed antioxidant activity both *in vitro* DPPH scavenging activity and reducing power and *in vivo* hemolysis induced by H_2_O_2_ in mice was reduced	Raman et al., 2016
	Freeze dried powder	*M. alba*/fruit	Freeze dried *M. alba* fruit resulted in increased activity of SOD and GSH-Px activity and lipid peroxidation was reduced in HFD induced wistar rats	Yang et al., 2010
	Methanol	*M. alba*/leave	Isolated astragalin showed strong prevention effect against free radical-induced oxidative hemolysis of human red blood cells and GSH depletion in RBCs	Choi et al., 2013
	Aqueous	*M. alba*/leaves	Separated flavonoids exhibited peroxyl radical-scavenging capacity and CAC against 2, 2′-Azobis (2-amidinopropane) dihydrochloride (AAPH) and Cu^2+^induced oxidative stress in HepG2 cells	Kim and Jang, 2011
Anti-diabetic	Ethyl acetate	*M. alba*/fruit	Soluble extract of *M. alba* fruit decrease fasting blood glucose (FBG) FBG and glycosylated serum protein (GSP) in STZ-induced diabetic mice	Wang et al., 2013
	Aqueous	*M. alba*/leave	*M. alba* leave decoction decreased blood glucose levels, inhibited hepatic glycogen loss, and prevented potential histopathological alterations in the pancreas and kidneys in STZ induced brown rat	Khyade and Hershko, 2018
	Ethanol	*M. alba*/stem bark	Significant alterations in glutathione and insulin level and blood glucose level was observed in STZ induced diabetic rats	S ALAnazi et al., 2017
	Hydroalcoholic	*M. alba*/leave	Chlorogenic acid and rutin responsible for anti-diabetic effect in STZ induced newborn Sprague-Dawley rats	Hunyadi et al., 2012
	Ethanol	*M. alba/*branches	Oxyresveratrol significantly reduced FPG in STZ-induced diabetic ICR mouse	Ahn et al., 2017
	Ethanolic	*M. nigra*/leave	Ethanolic extract may aid in preventing liver and kidney tissue damage in STZ induced rats	Hago et al., 2019
	Powdered leaf (mixed with diet)	*M. indica*/leave	Decrease in lipid peroxidation and the activity of CAT in erythrocytes in STZ induced albino rats	Andallu and Varadacharyulu, 2003
	Ethanol	*M. alba/*fruit	Polysaccharides like arabinose, galactose, and glucose exhibited excellent *invitro* hypoglycaemic effects	Chen et al., 2016
	Hydroalcoholic	*M. multicaulis*/ branch bark	Significant inhibition in activity α-glycosidase was observed *in vitro* and regulation of mRNA expression of glycometabolism genes including glucose-6-phosphatase (G6Pase) and glucokinase (GCK) in STZ induced diabetic mice	Liu et al., 2014
	Hydroalcoholic	*M. alba*/fruit	Anthocyanins have protection effect against β-cell damage in carboxy methyl cellulose treated Zucker diabetic fatty rats	Sarikaphuti et al., 2013
Anti-microbial	Ethanol	*M. alba*/leave	Purified and isolated DNJ inhibited the overgrowth and biofilm formation of *S. mutans*	Islam et al., 2008
	Methanol	*M. nigra*/leave	Significant anti-microbial and antioxidant properties, by the ability to increase antioxidant levels was observed against some pathogens.	Zhou et al., 2019
	Ethanolic	*M. alba/*leave	Inhibition against large population of pathogens	De Oliveira et al., 2015
	Ethanolic	*M. nigra*/stem bark and wood	Oxyreversterol, moracin, morusin, kuwanon isolated from wood and stem bark exhibited anti-microbial against some pathogens	Mazimba et al., 2011
	Aqueous, hydroalcoholic and methanol	*M. alba*/fruit	It showed inhibitory effect against some pathogens	Dimitrijević et al., 2014
	Methanol	*M. alba/*root	Kuwanon G possessed antibacterial activity against some oral pathogens	Park et al., 2003
	Methanol	*M. alba/*root	Kuwanon L, sanggenons B, C, D, G, moracin P, and sanggenol A, showed potential anti-microbial activities against *Bacillus subtilis* and *Escherichia coli*	Ristivojević et al, 2019
	Ethyl acetate	*M. alba*/twig	Isolated oxyresveratrol exhibit inhibitory effect against *Trichophyton rubrum* with the minimum inhibitory concentration of 1 mg/mL	Lu et al., 2017
	Methanol	*M. alba*/ leave	Isolated compounds chalcomoracin and moracin C inhibited the growth of *S. aureus*	Kim et al., 2012
	Ethanol	*M. alba*/leave	*M.* *alba* possess excellent antibacterial activity against periodontal disease	Gunjal et al., 2015
Hyperlipidemia	Freeze-dried powder	*M. alba*/fruit	Significant decrease in the atherogenic index and decrease in liver TG, TC and LDL in wistar rats fed with HFD	Yang et al., 2010
	Methanol	*M. alba*/root bark	Isolated compounds Albanol A and Albanol B significantly reduce in resistance towards major atherogenic modifications was observed in HFD fed hypercholesterolemia wistar rats	El-Beshbishy et al., 2006
	Ethanol	*M. alba*/root	Purified stilbenoids decrease in serum lipids, coronary artery risk index, and atherogenic index in high-cholesterol diet-induced hyperlipidemia Sprague Dawley rats	Jo et al., 2014
	Ethanol	*M. alba*/fruit	Significantly ameliorated LXRa-mediated lipogenesis and hepatic fibrosis markers such as smooth muscle actin in HFD induced obesity in C57BL/6 mice	Ann et al., 2015
	Aqueous	*M. alba* leaves	*M. alba* (1%) for twelve weeks might help prevent atherosclerosis involving the underlying mechanism of its anti-oxidative activity	Harauma et al., 2007
	Ethanol	*M. alba*/leave	*M. alba* resulted in a decrease in TG, TC, and LDL in triton WR-1339 induced hyperlipidemic ICR mice	Chen and Li, 2007
	Aqueous	*M. alba*/leave	Decrease in body weight and adipose tissue mass in HFD fed mice was observed	Lee et al., 2008
Anti-inflammatory	Hydroalcoholic	*M. nigra/*fruit	Secondary metabolites significantly decreased the number of leukocytes in the bronchoalveolar lavage fluid and serum levels of TNF	De Pádua Lúcio et al., 2018
	Methanol	*M. alba* root bark	Purified compounds albanol B, sanggenon B and sanggenon D exhibited inhibitory effects on NO production in LPS-stimulated RAW264.7	Wu et al., 2020
	Ethanol	*M. alba* fruit	*M. alba* fruit at a dose of 100 mg/kg body weight improves the learning and spatial memory in APP/PS1 transgenic mice	Liu and Du, 2020
	Ethanol	*M. alba* fruit	Phenolic compound has a positive effect on neuroprotection in AD	Qiao et al., 2015
	Ethanol	*M. alba/*stem	Morus in at a dose of 5 and 10 mg/kg delayed onset of convulsion and significantly increased level of brain GABA	Gupta et al., 2014
	Methanol	*M. atropurpurea*, *M. bombycis* and *M. alba*/branch	Bioactive constituent oxyresveratrol, is involved in the inhibition of CXCR-4-mediated chemotaxis and MEK/ERK pathway in T cells	Chen et al., 2013
	Methanol	*M. alba*/twig	Isolated compounds apigenin, albanin D, morachalcone A and mulberranol reduced the expression of reduced LPS iNOS and LPS-induced expression of COX-2 protein	Tran et al., 2017
	Ethanol	*M. alba*/stem	*M. alba* stem at a particular concentration may significantly suppress *P. gingivalis* LPS-induced IL-6 and IL-8 mRNA and protein expression	Yiemwattana et al., 2018
	Ethanol	*M. alba*/stem	Inhibition of the expression of COX- 2 mRNA and iNOS protein expression using RTPCR in LPS-induced RAW264.7cells	(Yiemwattana et al., 2018)
Anti-cancerous	Methanol	*M. alba/*leave	Morin extracted from methanolic extract of mulberry leaves exert an anti-cancerous potential in HeLa with an IC_50_ of 214.28 μM	Zhang et al., 2018
	Ethanol	*M. fructus/*leave	Oral administration of ethanolic extract in Balb/c nude mouse with subcutaneous U87MG glioma cells reduced tumor volume	Jeong et al., 2010
	Aqueous	*M. alba* leave	Preventive effect on obesity-mediated liver cancer in HepG2 cell proliferation	Chang et al., 2018

### 6.1 Anti-microbial properties

To date, a significant amount of research related to the anti-microbial properties of natural plants and their associated components is reported. Compound viz chalcomoracin with anti-microbial activity against methicillin-resistant *Staphylococcus aureus* (*S. aureus*) was isolated from *M. alba* leaves (Fukai et al., 2005). In another study, hydro-methanolic extract of stem bark of *M. alba* exhibited anti-microbial activity against *Enterococcus faecalis,* (*E. faecalis*), *Escherichia coli* (*E. coli*) *S. aureus, Staphylococcus epidermidis* (*S. epidermidis*) and *Salmonella typhimurium* (*S*. *typhimurium*) (Thabti et al., 2014). Budiman et al. 2017b, reported that the ethanolic extract of *M. nigra* was effective and inhibited the growth of *S. epidermidis* and *Propionibacterium acnes* (*P. acnes*) bacteria*.* Morin an anti-bacterial compound was isolated from *M. alba* fruits by LH-20 column chromatography and the structure was elucidated by ^13^C NMR and ^1^H NMR spectroscopy. The isolated compound showed modest anti-bacterial activity against *Streptococcus mutans* (*S*. *mutans*) at 5 and 2 mg/disc (Yang and Lee, 2012).

Apart from leaves, fruits, stems, and bark of Morus varieties, value-added products from them also exhibited potential anti-microbial activities. *M. nigra* juice exhibited anti-microbial potential against bacteria *Bacillus spizizenii (B. spizizenii*) and *Pseudomonas aeruginosa* (*P*. *aeruginosa*) with an inhibition zone of 19.68 mm and 19.87 mm, respectively (Khalid et al., 2011). Vinegar produced from *M. alba* showed antibacterial effect against *S. aureus, S. pyogenes*, *E. coli*, *E. faecalis, Erwinia carotovora* (*E. carotovora*) *Klebsiella oxytoca* (*K. oxytoca*), *Bacillus cereus* (*B. cereus*) and *Bacillus subtilis* (*B. subtilis*)*,* anti-fungal activities against *Candida albicans* (*C. albicans*). Among all bacteria, *S. aureus* (28 mm) followed by *S. pyogenes* (20.6 mm) exhibited the highest zone of inhibition while *E. coli* showed the lowest zone of inhibition 5.3 mm and for *C. albicans* 9.6 mm zone of inhibition was observed (Karaagac et al., 2016).

### 6.2 Anti-hyperglycaemic property

Diabetes mellitus (DM) is now considered the third most life-threatening metabolic condition in the world, characterized by hyperglycaemia (high blood glucose levels) (Wang et al., 2013). There are more than 170 million people affected by this chronic disease globally and it is estimated to rise by 50 percent by the year 2030, with the highest increase forecasted in developing countries like Asia, Africa, and South America.

Various studies have reported the anti-diabetic properties of mulberry. The leaves of *M. alba* have been included in Chinese traditional medicine since a long time for treatment and prevention of diabetes due to the presence of functional chemical constituents that suppress raised blood sugar levels following a carbohydrate-rich meal (Miyahara et al., 2004). Polysaccharides in *M. alba* fruit hold excellent potential for anti-diabetic activity. A significant reduction in fasting serum insulin, homeostasis model of assessment-insulin resistance, fasting glucose level, glycated serum protein, and repairment of impaired pancreatic tissues of the diabetic rats was observed after seven weeks of treatment with *M. alba* fruit polysaccharides (two fractions)in wister rats (Jiao et al., 2017). Extracted DNJ appreciably decreased blood glucose and insulin levels, reversed insulin resistance, and enhanced serum lipid levels and in high fat diet-induced (HFD) diabetic Kunming mice (Hu et al., 2019). A single dose of *M. alba* leaves extract with a DNJ concentration of 3, 6, or 9 mg was given to subjects with fasting glucose levelsof100-140 mg/dL. Meanwhile, 6 mg of DNJ for twelve weeks was given to subjects with a fasting glucose level of 110–140 mg/dL. Long-term ingestion of DNJ rich *M. alba* leaves extract resulted in improved post-prandial glycemic control in patients with damaged glucose metabolism (Asai et al., 2011). Ingestion of diet containing *M. alba* extract repeatedly may maintain postprandial glucose levels. In a study after eight weeks of repeated ingestion of *M. alba* extract in KK-Ay mice, fasting plasma glucose (FPG) and insulin levels were measured and found an appreciable reduction in insulin resistance, and the onset time of urinary glucose excretion was delayed (Tanabe et al., 2011).

### 6.3 Anti-hyperlipidemic activity

Obesity is defined as an unusual deposition of fat that extents risk to health. It is one of the serious concerns prevailing today across the globe as it increases the risk of diabetes, heart disease, and cancer. Beneficial phytoconstituents in mulberry has increased the attention of researchers to explore its best potential for anti-obesity characteristics.

The effect of *M. alba* fruit on the lipid profile of humans in the age group of 30–60 years was studied. After consumption of *M. alba* fruit for six weeks at a dose of 45 g per day, a significant decrease in the total cholesterol (TC) and low-density lipoprotein (LDL) levels in blood was noted (Sirikanchanarod et al., 2016). Similarly, in another study, on oral administration of DNJ rich *M. alba* leaves extract at a dose of 12 mg three times per day before meals reduced the level of serum triglycerides (ST) and lipoproteins. DNJ, extracted from *M. alba* leaves when administered to diet-induced obese mice, was found beneficial for lowering down the levels of plasma triacylglycerol besides activating the β-oxidation system and reducing the lipid accumulation in the liver (Tsuduki et al., 2013). It may also reduce hyperlipidemia by moderating feeding behaviour and endoplasmic reticulum stress in the hypothalamus of mice with HFD obesity in C57BL/6J mice (Kim et al., 2017). Mulberry juice in combination with blueberry juice may aid in decreasing the blood cholesterol, resistance to insulin and leptin secretin attenuated lipid accumulation in HFD-induced obesity C57BL/6 mice (Wu et al., 2013). Isolated oxyresveratrol from *M. alba* wood monitored the degradation of fatty acids and hepatic lipogenesis to ameliorate non-alcoholic fatty liver fat in HFD mice (Lee et al., 2018). In another study, it was observed that administration of aqueous extract of *M. alba* fruit for twelve weeks in male Syrian golden hamsters resulted in lowered cholesterol, free fatty acid, and hepatic lipids (Peng et al., 2011).

Recently extract of leaves of *M. alba* fermented with 10% of *Cordyceps militaris* significantly stimulated the lipolysis of primary adipocytes at a suitable concentration and hence can be utilised as lipolytic agents to treat obesity (Lee et al., 2020).

### 6.4 Anti-inflammatory activity

The presence of microorganisms (bacteria, viruses, and fungi) in specific tissues and their circulation in the blood can cause one of the complex vascular biological responses called inflammation. It can damage the body if not regulated after a certain period and may cause some chronic diseases like rheumatoid arthritis, cardiovascular diseases, and cancers. Studies have highlighted that regular consumption of natural plants with anti-inflammatory activities can help in the treatment of acute chronic inflammation.

Recently novel extraction technique, high hydrostatic pressure was employed as an extraction method in *M. alba.* High hydrostatic pressure extracts inhibited *in vitro* release of nitric oxide (NO) and messenger ribonucleic acid (mRNA) expression of nitric oxide synthase 2(NOS2) and reduction of cytokines such as interleukin (IL)-6 and tumor necrosis factor (TNF)-α, which are associated with inflammation in lipopolysaccharide (LPS)-induced RAW264.7 cells (Jung et al., 2019). Similarly, methanolic extract of *M. alba* root bark blocked NO production through suppressing inducible nitric oxide synthase (iNOS)over-expression in LPS-stimulated RAW264.7 cells (Eo et al., 2014).

Ethanolic extract of *M. alba* leaves effectively reduces pro-inflammatory mediators and cytokine production by modulating the LPS-induced activation of macrophage cells by suppressing nuclear factor-κB (NF-κB) activation (Park et al., 2013). Ethanolic extract of stem of *M. alba* at a concentration of 20 and 40 µg/ml show anti-inflammatory activity in LPS-stimulated RAW264.7 macrophage cell line by inhibition of NO production via suppression of both the protein and iNOS mRNA.

Anthocyanins in *M. nigra* fruits have been reported to have anti-inflammatory activity. C3G and C3R exert an anti-inflammatory effect through inhibition of pro-inflammatory cytokine in xylene-induced ear edema and carrageenan-induced paw edema in mice (Chen et al., 2016). Aqueous extract of *M. alba* root has strong anti-histamine and anti-allergic activity by inhibiting compound 48/80-induced systemic allergic reaction and histamine release *in vitro* and *in vivo*. Mast cell-mediated type allergic reactions are also inhibited by the root extract of *M. alba* (Chai et al., 2005).

Hence, Morus species can be a budding natural source of anti-inflammatory drugs.

### 6.5 Anti-cancer action

Cancer, with different forms, is believed as one of the primary fatal diseases prevailing globally and the rate is surpassing with a good number. Recently, many types of cancers have been considered to be among the group of diseases that are common reason for death. Many medications are available in the market to treat different forms of cancer, but successful and safe drugs are rarely available. As compared to synthetic and semi-synthetic compounds, naturally occurring bioactive compounds particularly phenolic compounds are less toxic and safer (Habauzit and Morand, 2012).

**Fig. 4:**
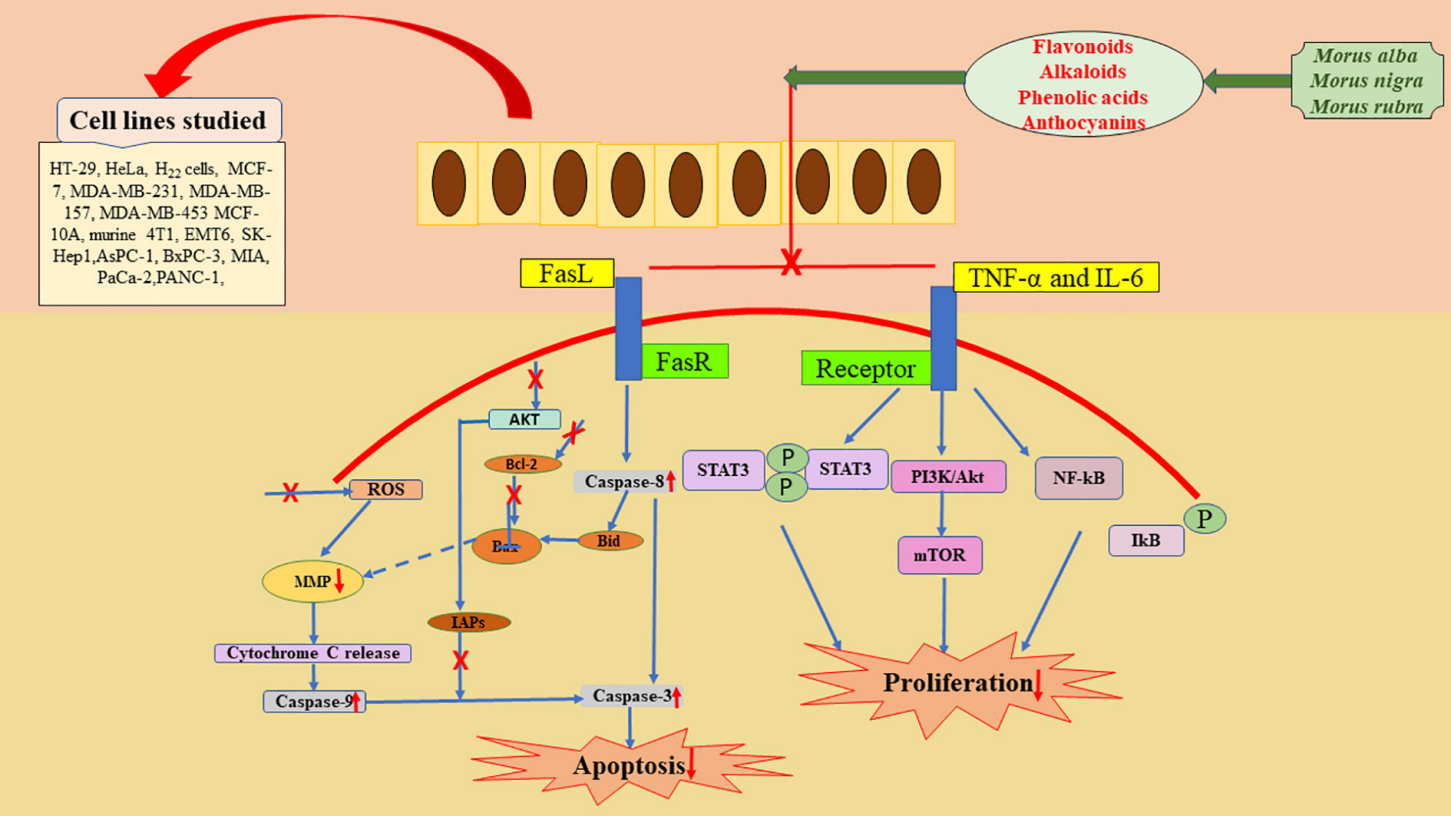
Mechanism of anti-cancer activity of mulberry.

Many naturally occurring substances exert their anti-cancer activity through apoptosis of tumor cells and by arresting the cell cycle, which is thought to be the best way to avoid or fight irregular cell growth (Dewanjee et al., 2017). Detailed mechanism of anti-cancer activity of Morus species is mentioned in Fig. 4**.** Traditionally, medicine value of the mulberry plant is known to humans from the earliest ages. Clinically, mulberry plant has the potential of inhibition of cell proliferation most likely due to the presence of flavonoids that are highly effective against certain types of cancers. Some studies reported the role of mulberry in cancer prevention in animal models. Purified anti-proliferative lectin from *M. alba* leaves induced cell death through apoptosis in human breast cancer (MCF-7) and colon cancer (HCT-15) cells by inducing essential morphological changes and DNA fragmentation related to apoptosis (Deepa et al., 2012). The root bark of *M. alba* contains flavanone glycoside, 5,2′-di-hydroxyflavanone-7,4′-di-O-β-D-glucoside (steppogenin-7,4′-di-O-β-D-glucoside) has anti-proliferation activity against HO-8910 cells in human ovarian cancer (Zhang et al., 2009).

### 6.6 Neurodegenerative actions

Plants play an important role in treating cognitive disorders. Many medicinal plants exhibit an essential role in the treatment and prevention of numerous neuronal dysfunctions and neurodegenerative diseases. There are numerous studies reported on natural medicinal plants where the isolated bioactive constituents such as polyphenolics and alkaloids can considerably delay neurodegeneration and may improve cognitive function and memory (Mohebbati et al., 2017). Lyophilised ethanolic extract of mulberry fruit protects neuronal cells against oxidative stress-induced apoptosis through the enhancement of production of antioxidant enzymes and brain-derived neurotrophic factor formation by stabilizing the activation of the TrkB/Akt pathway in swiss CD-1 mice (Shin et al., 2019).

The role of antioxidants in *M. alba* fruits is well documented in many studies. Non-anthocyanins in *M. alba* fruit like rutin and quercetin have neuroprotective effects besides having multi-bioactive functions (Shih et al., 2010, Isabelle et al., 2008, Zhang et al., 2009). These non-anthocyanins have an impact on Parkinson's disease (PD) models. Effect of ethanolic extract of *M. alba* fruit in *in vitro* and *in vivo* models of PD was studied on dopaminergic neuron protection using the SH-SY5Y neuroblastoma stressed with 6-hydroxydopamine (6-OHDA) and mesencephalic dopamine neurons stressed with 6-OHDA and 1-methyl-4-phenylpyridinium (MPPþ). The effect of the same extract was also studied on *in vivo* models, where PD was induced by 1-methyl-4-phenyl-1,2,3,6-tetra-hydropyridine (MPTP). Symptoms of PD like bradykinesia and MPTP-induced dopaminergic neuronal damage in an immunocytochemical analysis of the substantia nigra pars compacta (SNpc) and striatum (ST) was prevented (Kim et al., 2010).

## 7 Miscellaneous activities of Morus species

In traditional medicine, the usage of *M. alba* is credited toits excellent diuretic properties, which are mainly owed to the free radical scavenging attribute. A mixture made of *M. alba* fruit (ethanolic extract) and gentamicin with a dosage of 200 mg/kg/day and 80 mg/kg/day, respectively for three weeks controlled the serum uric acid, creatinine, blood urea nitrogen content in rabbits (Ullah et al., 2016). Mulberroside A (stilbene glycoside) from twigs of *M. alba* at 10, 20, and 40 mg/kg significantly treated renal dysfunction by suppressing the elevated protein and mRNA levels of renal glucose transporter 9(mGLUT9) and urate transporter 1 (mURAT1) in hyperuricemia mice (Wang et al., 2011). Morusinol a flavonoid extracted from the root bark of *M. alba* may significantly inhibit arterial thrombosis that was recently studied for cardiovascular potential (Lee et al., 2012). The alcoholic extract of *M. alba* leaves showed hepatoprotective effect against hepatotoxicity induced by carbon tetrachloride and paracetamol in Swiss albino mice (Hogade et al., 2010). *M. alba* juice is helpful in the prevention of food-borne viral infection (norovirus infection) by inhibiting the internalization and replication of murine norovirus-1 (MNV-1), wherein it may affect the adherence or internalization of feline calicivirus-F9 (FCV-F9) virions (Lee et al., 2014). *M. alba* juice may also induce anti-stress activity in Balb/c mice through a mechanism of radical scavenging activity (Sakagami et al., 2006).

From current scientific studies, it can therefore be inferred that mulberry exhibits substantial antioxidant capacity *in vitro* and *in vivo*, making them promising nutraceuticals.

## 8 Conclusion

Natural products are now being re-emphasized in order to address a variety of health issues. The correlation between health and diet is well established, and consumers are becoming more conscious of their eating habits. Investigating these connections has resulted in the creation of functional, nutraceuticals, and pharma foods, which are now dominating the global nutrition market. The current review intended to highlight the significance and application of Morus species in different areas and it becomes amply clear from the above discourse that mulberry is a versatile medicinal plant with enormous vitality. Recent approaches regarding the functional applications revealed that Morus species and their bioactive phytochemicals display a wide variety of biomedical activities, including antioxidants, anti-diabetic, hypo-lipidemic, anti-obesity, anti-hypertensive, and anti-atherosclerosis, etc. Morus extracts or their constituents particularly flavonoids like chlorogenic acid, quercetin, rutin and isoquercitrin scavenge free radicals exhibiting potential against oxidative stress. Alkaloids like DNJ and fagomine present in *M. alba* exhibit potential glucosidase inhibition. Compounds like moracin, morusin, kuwanon isolated from wood and stem bark of *M. nigra* exhibit anti-microbial activity. Tyrosinase inhibition properties of Morus species are comparable to kojic acid that makes it a wonderful ingredient in cosmetics. Chemical compounds like maclurin and morin, mulberroside F, oxyresveratrol and austraone isolated from different parts of *M. alba* exhibit potential tyrosinase inhibition activity. In addition to its exceptional usage as a nutraceutical in the pharmaceutical industry, it is often used in food industries because of the power source of anthocyanins that exhibit strong antioxidant properties and are used as a natural colouring agent. Being low in calories, this plant can be utilised in the formulation of hypocaloric foodstuffs and can be added as a novel ingredient to enhance the functional properties of existing foods. Value added products like jam, jelly, wine, vinegar, tea, syrup, squash and many more are formulated from Morus that aids industrialists for effective utilization of its fruits and leaves. Additionally mulberry is among the major ingredients in many traditional formulations sold worldwide. Chemical composition of mulberry is already extensively studied, there are still some unidentified biological compounds that require proper exploration. It is necessary, however, to investigate the metabolites produced *in vivo* and how they exert their biological effects in future studies.

## Declaration of Competing Interest

The authors declare that they have no known competing financial interests or personal relationships that could have appeared to influence the work reported in this paper.
